# Egyptian Pad

**Published:** 1880-12

**Authors:** 


### EGYPTIAN PAD.

Gum galbanum, ground, one-half ounce; gum myrrh, ground one-quarter ounce; gum camphor, ground, one drachm; gum ammoniac, ground, one-half ounce; gum benzoin, ground, one-quarter ounce; sandalwood, ground, two drachms. Mix them. If the gums are too moist to admit of reduction, dry them with the aid of heat. The quantity given is for one pad. It should be worn over the sternum, about midway between the heart and liver. These pads are said to prevent and cure fever and ague and all malarious diseases.—*Copied from Nelson's "Hand Book of Private Formulas."*

